# Picornavirus Salivirus/Klassevirus in Children with Diarrhea, China

**DOI:** 10.3201/eid1608.100087

**Published:** 2010-08

**Authors:** Tongling Shan, Chunmei Wang, Li Cui, Ying Yu, Eric Delwart, Wei Zhao, Caixia Zhu, Daoliang Lan, Xiuqiang Dai, Xiuguo Hua

**Affiliations:** Shanghai Jiao Tong University, Shanghai, People’s Republic of China (T. Shan, C. Wang, L. Cui, Y. Yu, W. Zhao, C. Zhu, D. Lan, X. Dai, X. Hua); Huazhong Agricultural University, Wuhan, People’s Republic of China (C. Wang); Blood Systems Research Institute, San Francisco, CA, USA (E. Delwart); University of California, San Francisco (E. Delwart); 1 These authors contributed equally to this article.

**Keywords:** Salivirus/Klassevirus, diarrhea, China, viruses, dispatch

## Abstract

To learn more about salivirus/klassevirus, we tested feces of children with diarrhea in China during 2008–2009. We isolated the virus from 9/216 diarrhea samples and 0/96 control samples. The nearly full polyprotein of 1 isolate, SH1, showed 95% identity with a salivirus from Nigeria, indicating widespread distribution and association with diarrhea.

Diarrhea causes ≈2 million deaths each year (*1*), primarily among young children in developing countries (*1**,**2*). The causative agents for ≈40% of cases remain unknown (*2**–**4*).

Studies have documented an association between Aichi viruses and gastroenteritis (*5**,**6*). Recent studies have documented human infections with the salivirus/klassevirus-related Aichi virus (*7**–**9*) that were associated with diarrhea (*9*). The previously unknown picornavirus klassevirus has recently been recently detected in fecal samples from persons with diarrhea in the United States and Australia and in sewage in Spain (*7**,**8*). Closely related saliviruses have been identified in fecal samples from persons in Nigeria, Tunisia, and Nepal and have been statistically associated with diarrhea in Nepal (*9*).

Klassevirus/salivirus is genomically organized similar to other picornaviruses and most closely related to *Aichi virus* in the genus *Kobuvirus* (*5*–*7*). The family *Picornaviridae* is highly diverse and contains small, nonenveloped viruses with a single-stranded positive-sense RNA genome that encodes a single polyprotein; it consists of 12 genera and 2 possibly new genera (*7*), a subset of which can infect and cause disease in humans.

To our knowledge, there have been no reports of infection with this virus in the People’s Republic of China. Therefore, to extend these initial findings, we tested for this newly characterized virus in fecal samples from children with diarrhea in China and sequenced the nearly full genome of 1 isolate, SH1.

## The Study

During April 2008–March 2009, a total of 216 fecal samples were collected from children, 2–6 years of age, who were hospitalized with diarrhea in Shanghai Children’s Hospital, China. A total of 96 children, 3–5 years of age, from 2 childcare centers in Shanghai City were included as healthy controls.

Samples were suspended to 10% (wt/vol) in phosphate-buffered saline (0.01 M, pH 7.4), and total RNA was extracted from 200 µL of the suspension by using TRIZOL reagent (Invitrogen, Carlsbad, CA, USA). Viral RNA was dissolved in 30 µL RNase-free water and stored at –80°C.

To understand the possible association between salivirus/klassevirus and diarrhea, we conducted prevalence studies by using nested reverse transcription–PCR (RT-PCR). We used a nested set of PCR primers (SAL-L1, 5′-CCCTGCAACCATTACGCTTA-3′; SAL-R1, 5′-CACACCAACCTTACCCCACC-3′; SAL-L2, 5′-ATTGAGTGGTGCAT(C)GTGTTG-3′; SAL-R2, 5′-ACAAGCCGGAAGACGACTAC-3′) to amplify a 414-bp fragment located in the 5′ untranslated region (UTR). The expected size DNA bands were excised from an agarose gel, purified with the AxyPrep DNA gel extraction kit (Axygen, Union City, CA, USA), cloned into pMD-18T vector (TaKaRa, Shiga, Japan), and sequenced on an Applied Biosystems 3730 DNA Analyzer (Invitrogen). Of 216 samples, 9 (4.2%) were positive for the newly described picornavirus; ages of the children were 2 years (n = 1), 3 years (n = 3), 4 years (n = 2), 5 years (n = 1), and 6 years (n = 2). Sequence analysis, based on the 414-bp sequences, showed that these 9 sequences shared 98.3%–99.8% identity with each other, suggesting that they could be considered members of the same virus species. The sequences shared 94.7%–97.3% sequence identities with GenBank isolates nos. GQ253930 (klassevirus 1, Australia), GQ184145 (human klassevirus 1, USA), and GQ179640 (salivirus, Nigeria). The 9 salivirus/klassevirus–positive samples were further investigated for Aichi virus, parechovirus, norovirus, sapovirus, rotavirus, astrovirus, and cosavirus by using RT-PCR with the primers previously described (*10**–**13*). Results indicated that 1 sample, for which the 518-bp–specific fragment was sequenced, was also positive for human parechovirus. No salivirus/klassevirus was detected in samples from the 96 healthy controls. The Fisher exact test showed a significant (p = 0.03) association between salivirus/klassevirus detection and diarrhea.

The complete genomic sequence of strain SH1 was then determined by using 10 sets of specific oligonucleotide primers designed on the complete genome of GQ184145, GQ253930, and GQ179640. The nearly full-genome genome of this virus strain was 7,798 nt and contained an open reading frame (ORF) with a length of 7,107 nt, encoding a putative polyprotein precursor of 2,369 aa. This ORF is preceded by a 5′ UTR at least 624 nt long and followed by a 3′ UTR at least 67 nt long. Phylogenetic analysis using the more variable P1 region of SH1 and 45 representative picornaviruses (including 3 salivirus/klassevirus strain) confirmed the close relationship of this strain with strains from other continents; it was most closely related to a human klassevirus from the United States (GQ184145) (Figure).

**Figure:**
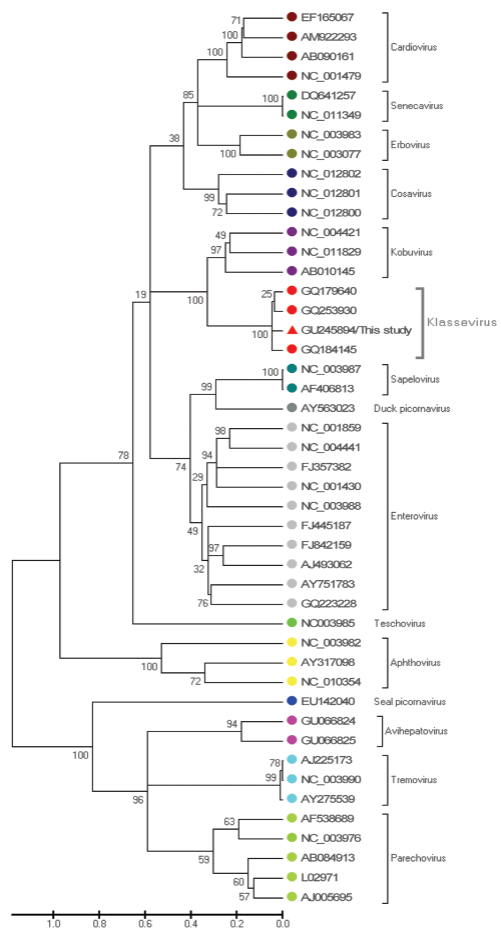
Phylogenetic analysis of the more variable P1 region of the salivirus/klassevirus isolated from fecal samples of 9 (4.2%) of 216 children with diarrhea in the People’s Republic of China, April 2008–March 2009, and 45 representative strains. Phylogenetic tree was constructed by the neighbor-joining method with 1,000 bootstrap replicates by using MEGA4.0 software (www.megasoftware.net). Bootstrap values are indicated at each branching point. The isolate SH1 is marked with a triangle. Scale bar indicates estimated phylogenetic divergence.

The nearly full genome of SH1 has been submitted to GenBank under accession no. GU245894. The 9 partial 414-bp sequences of salivirus/klassevirus are deposited in GenBank under accession nos. GU376738–GU376746.

## Conclusions

Our finding of salivirus/klassevirus in fecal samples of children with diarrhea in China is consistent with Li et al.’s report of this virus’ association with diarrhea (*9*). This finding, plus the identity with the Nigeria reference strain, support widespread distribution of this newly characterized virus species and its association with diarrhea**.**
